# 6-Oxo-5-[(trifluoro­meth­yl)sulfon­yl]-1,2,4a,5,6,11b-hexa­hydro-1,3-dioxolo[4,5-*j*]phenanthridin-2-yl benzoate

**DOI:** 10.1107/S1600536811001085

**Published:** 2011-01-15

**Authors:** Chunli Wu, Pan Li, Xiufang Shi, Xiaotao Pan, Jizhou Wu

**Affiliations:** a School of Pharmacy, Tongji Medical College, Huazhong University of Science and Technology, Wuhan 430030, People’s Republic of China; b School of Pharmaceutical Sciences, Zhengzhou University, Zhengzhou 450001, People’s Republic of China

## Abstract

In the title compound, C_22_H_16_F_3_NO_7_S, the two benzene rings are almost perpendicular, the dihedral angle between their mean planes being 87.1 (1)°. The terminal O atom of the benzoate moiety is disordered over two positions with site occupancies of 0.244 (15) and 0.756 (15). The crystal structure is stablized by two types of weak inter­molecular C—H⋯O hydrogen bonds.

## Related literature

The title compound is an unexpected product in our recent synthesis route of phenanthridones alkaloids. It shows potent inhibitory activity against the MCF-7 cells, SK—N—SH cells and SPC-A-1 cells. For details of the synthesis, see: Banwell *et al.* (1995 ▶); Szántó *et al.* (2009*a* ▶,*b* ▶); Pampin *et al.* (2003 ▶). For a recent study on the anti­tumor activity of phenanthridones alkaloids, see: Matveenko *et al.* (2009 ▶).
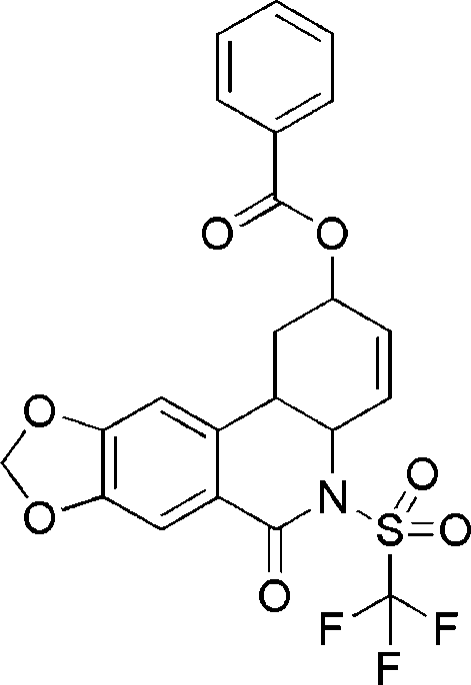


## Experimental

### 

#### Crystal data

C_22_H_16_F_3_NO_7_S
*M* *_r_* = 495.42
Triclinic, 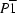

*a* = 5.3521 (5) Å
*b* = 15.5146 (16) Å
*c* = 15.5615 (14) Å
α = 114.351 (2)°
β = 95.145 (1)°
γ = 97.072 (1)°
*V* = 1154.07 (19) Å^3^
*Z* = 2
Mo *K*α radiation
μ = 0.21 mm^−1^
*T* = 298 K
0.45 × 0.33 × 0.19 mm

#### Data collection

Bruker SMART CCD area-detector diffractometer
Absorption correction: multi-scan (*SADABS*; Sheldrick, 1996 ▶) *T*_min_ = 0.913, *T*_max_ = 0.962
5980 measured reflections
4008 independent reflections
2079 reflections with *I* > 2σ(*I*)
*R*_int_ = 0.041

#### Refinement

*R*[*F* ^2^ > 2σ(*F* ^2^)] = 0.066
*wR*(*F* ^2^) = 0.163
*S* = 1.00
4008 reflections
312 parameters
H-atom parameters constrained
Δρ_max_ = 0.41 e Å^−3^
Δρ_min_ = −0.30 e Å^−3^

### 

Data collection: *SMART* (Siemens, 1996 ▶); cell refinement: *SAINT* (Siemens, 1996 ▶); data reduction: *SAINT*; program(s) used to solve structure: *SHELXS97* (Sheldrick, 2008 ▶); program(s) used to refine structure: *SHELXL97* (Sheldrick, 2008 ▶); molecular graphics: *SHELXTL* (Sheldrick, 2008 ▶); software used to prepare material for publication: *SHELXTL*.

## Supplementary Material

Crystal structure: contains datablocks I, global. DOI: 10.1107/S1600536811001085/zq2082sup1.cif

Structure factors: contains datablocks I. DOI: 10.1107/S1600536811001085/zq2082Isup2.hkl

Additional supplementary materials:  crystallographic information; 3D view; checkCIF report

## Figures and Tables

**Table 1:** Hydrogen-bond geometry (Å, °)

*D*—H⋯*A*	*D*—H	H⋯*A*	*D*⋯*A*	*D*—H⋯*A*
C3—H3⋯O4^i^	0.93	2.51	3.341 (5)	149
C15—H15*A*⋯O2^ii^	0.97	2.48	3.202 (5)	131

Symmetry codes: (i) 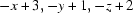; (ii) 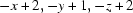.

**Fig. 1.**
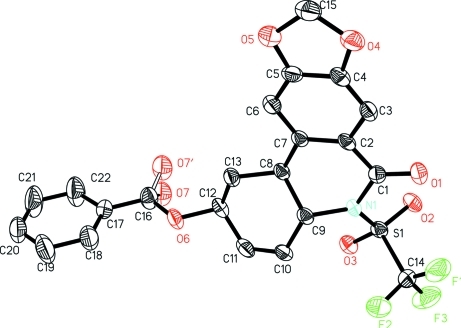
The molecular structure of the compound, with atom labels and 30% probability displacement ellipsoids for non-H atoms.

**Fig. 2.**
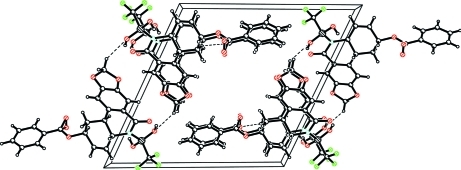
Packing diagram.

## supplementary crystallographic information

### Comment

This paper shows an unexpected product of our study about synthesis and structural characterization of phenanthridones alkaloids. The title compound was obtained under Banwell modification of the Bischler-Napieralski reaction (Banwell *et al.*, 1995) of 1-benzoyloxy-4-(methoxycarbonylamino)-5-(3,4-methylenedioxyphenyl)cyclohex-2-ene. The formation of this product is probably due to the overuse of Tf_2_O (Pampin *et al.*, 2003), which is confirmed when a smaller amount of Tf_2_O is used. What makes us excited is that the unexpected product shows potent inhibitory activity (Matveenko *et al.*, 2009) against the MCF-7 cells (IC50 = 4.46µg/ml), SK—N—SH cells (IC50 = 1.89µg/ml) and SPC-A-1 cells (IC50 = 1.35µg/ml).

In the crystal structure of the title compound, the two benzene rings are almost perpendicular, the dihedral angle between the mean planes of the rings is 87.1 (1)°. The terminal O atom of the benzoate moiety is disordered over two positions with site-occupancies of 0.244 (15) and 0.756 (15). The environment of the N atom is essentially planar, and the bond angles C1—N1—C9, C9—N1—S1 and C1—N1—S1 around the N atom are 114.8 (3), 125.4 (2) and 119.7 (2)°, respectively. The molecules are linked into a framework by means of two types of weak C—H···O hydrogen bonds linking the CH_2_ group of the 1,3-dioxolane and one O atom of the sulfonyl group [H···O = 2.48 Å, C···O = 3.201 (6) Å and C—H···O = 131°] and one CH group of the C2-C7 cyclohexene ring and one O atom of the benzodioxole moiety [H···O = 2.51 Å, C···O = 3.342 (5) Å and C—H···O = 149°].

### Experimental

1-Benzoyloxy-4-(methoxycarbonylamino)-5-(3,4-methylenedioxyphenyl)cyclohex-2-ene (0.46 mmol) and 4-DMAP (1.38 mmol) were dissolved in dry CH_2_Cl_2_ (12 ml) and cooled to 0°C. To this mixture was added a solution of triflic anhydride (3.68 mmol) in dry CH_2_Cl_2_ (2 ml) over a period of 15 min. The mixture was stirred overnight at ambient temperature. The reaction mixture was then washed with saturated NaHCO_3_ solution, 1*M* hydrochloric acid and saturated NaHCO_3_ solution, subsequently. The organic layer was evaporated, and the product was isolated by column chromatography on silica (eluent: petroleum ether/acetone = 5:1). Crystals suitable for X-ray analysis were grown by slow evaporation from acetone-ethanol solution at room temperature for two weeks.

^1^H NMR (400 MHz, CDCl_3_, ppm): 8.11–8.04 (*m*, 2H), 7.65–7.56 (*m*, 2H), 7.47 (*t*, *J* = 7.7 Hz, 2H), 6.87 (*s*, 1H), 6.20 (*dt*, J = 10.6, 2.2 Hz, 1H), 6.09 (*s*, 2H), 6.01 (*dd*, *J* = 10.9, 0.9 Hz, 1H), 5.91 (*ddd*, *J* = 9.5, 5.5, 2.8 Hz, 1H), 4.67 (*ddd*, *J* = 10.6, 5.5, 2.7 Hz, 1H), 3.57–3.47 (m, 1H), 3.05 (*dd*, *J* = 13.5, 4.7 Hz, 1H), 1.73 (*td*, *J* = 12.9, 10.2 Hz,1*H*).

^13^C NMR (101 MHz, CDCl_3_, ppm): 165.96, 163.58, 153.84, 147.76, 138.98, 133.38, 129.72, 129.46, 128.49, 125.65, 121.61, 109.76, 104.44, 102.54, 69.06, 63.96, 39.83, 30.84.

### Refinement

All H atoms were placed geometrically and treated as riding on their parent atoms with C—H are 0.96 Å (methylene) or 0.93 Å (aromatic), 0.82 Å (hydroxyl)and *U*_iso_(H) =1.2*U*_eq_(C).

### Crystal data

**Table:**

C_22_H_16_F_3_NO_7_S	*Z* = 2
*M**_r_* = 495.42	*F*(000) = 508
Triclinic, *P*1	*D*_x_ = 1.426 Mg m^−^^3^
Hall symbol: -P 1	Melting point = 394–397 K
*a* = 5.3521 (5) Å	Mo *K*α radiation, λ = 0.71073 Å
*b* = 15.5146 (16) Å	Cell parameters from 1287 reflections
*c* = 15.5615 (14) Å	θ = 2.5–25.0°
α = 114.351 (2)°	µ = 0.21 mm^−^^1^
β = 95.145 (1)°	*T* = 298 K
γ = 97.072 (1)°	Needle, colorless
*V* = 1154.07 (19) Å^3^	0.45 × 0.33 × 0.19 mm

### Data collection

**Table:**

Bruker SMART CCD area-detector diffractometer	4008 independent reflections
Radiation source: fine-focus sealed tube	2079 reflections with *I* > 2σ(*I*)
graphite	*R*_int_ = 0.041
φ and ω scans	θ_max_ = 25.0°, θ_min_ = 2.5°
Absorption correction: multi-scan (*SADABS*; Sheldrick, 1996)	*h* = −6→6
*T*_min_ = 0.913, *T*_max_ = 0.962	*k* = −12→18
5980 measured reflections	*l* = −18→15

### Refinement

**Table:**

Refinement on *F*^2^	Primary atom site location: structure-invariant direct methods
Least-squares matrix: full	Secondary atom site location: difference Fourier map
*R*[*F*^2^ > 2σ(*F*^2^)] = 0.066	Hydrogen site location: inferred from neighbouring sites
*wR*(*F*^2^) = 0.163	H-atom parameters constrained
*S* = 1.00	*w* = 1/[σ^2^(*F*_o_^2^) + (0.0699*P*)^2^] where *P* = (*F*_o_^2^ + 2*F*_c_^2^)/3
4008 reflections	(Δ/σ)_max_ < 0.001
312 parameters	Δρ_max_ = 0.41 e Å^−^^3^
0 restraints	Δρ_min_ = −0.30 e Å^−^^3^

### Special details

**Table:**

Geometry. All e.s.d.'s (except the e.s.d. in the dihedral angle between two l.s. planes) are estimated using the full covariance matrix. The cell e.s.d.'s are taken into account individually in the estimation of e.s.d.'s in distances, angles and torsion angles; correlations between e.s.d.'s in cell parameters are only used when they are defined by crystal symmetry. An approximate (isotropic) treatment of cell e.s.d.'s is used for estimating e.s.d.'s involving l.s. planes.
Refinement. Refinement of *F*^2^ against ALL reflections. The weighted *R*-factor *wR* and goodness of fit *S* are based on *F*^2^, conventional *R*-factors *R* are based on *F*, with *F* set to zero for negative *F*^2^. The threshold expression of *F*^2^ > σ(*F*^2^) is used only for calculating *R*-factors(gt) *etc*. and is not relevant to the choice of reflections for refinement. *R*-factors based on *F*^2^ are statistically about twice as large as those based on *F*, and *R*- factors based on ALL data will be even larger.

### Fractional atomic coordinates and isotropic or equivalent isotropic displacement parameters (Å^2^)

**Table:**

	*x*	*y*	*z*	*U*_iso_*/*U*_eq_	Occ. (<1)
C1	0.9593 (7)	0.7149 (3)	1.0563 (3)	0.0459 (9)	
C2	1.0231 (6)	0.6470 (3)	0.9656 (3)	0.0419 (9)	
C3	1.1954 (7)	0.5870 (3)	0.9721 (3)	0.0493 (10)	
H3	1.2688	0.5923	1.0309	0.059*	
C4	1.2502 (7)	0.5211 (3)	0.8890 (3)	0.0494 (10)	
C5	1.1403 (8)	0.5127 (3)	0.8020 (3)	0.0535 (10)	
C6	0.9739 (8)	0.5702 (3)	0.7929 (3)	0.0562 (11)	
H6	0.9031	0.5633	0.7331	0.067*	
C7	0.9146 (7)	0.6395 (3)	0.8768 (3)	0.0428 (9)	
C8	0.7389 (7)	0.7092 (3)	0.8759 (2)	0.0443 (9)	
H8	0.5712	0.6850	0.8859	0.053*	
C9	0.8435 (7)	0.8052 (3)	0.9615 (2)	0.0447 (9)	
H9	1.0261	0.8191	0.9598	0.054*	
C10	0.7331 (8)	0.8881 (3)	0.9576 (3)	0.0548 (10)	
H10	0.7630	0.9463	1.0118	0.066*	
C11	0.5952 (8)	0.8810 (3)	0.8797 (3)	0.0629 (12)	
H11	0.5412	0.9360	0.8806	0.075*	
C12	0.5204 (8)	0.7912 (3)	0.7908 (3)	0.0546 (11)	
H12	0.3471	0.7607	0.7889	0.066*	
C13	0.7022 (7)	0.7217 (3)	0.7835 (2)	0.0516 (10)	
H13A	0.8653	0.7460	0.7719	0.062*	
H13B	0.6342	0.6600	0.7304	0.062*	
C14	0.8693 (10)	0.9435 (3)	1.2227 (3)	0.0640 (12)	
C15	1.3895 (9)	0.4010 (3)	0.7757 (3)	0.0738 (13)	
H15A	1.3118	0.3353	0.7593	0.089*	
H15B	1.5551	0.4007	0.7549	0.089*	
C16	0.3455 (10)	0.7706 (4)	0.6349 (3)	0.0699 (13)	
C17	0.3822 (9)	0.7953 (3)	0.5552 (3)	0.0614 (12)	
C18	0.5931 (11)	0.8560 (4)	0.5573 (3)	0.0960 (17)	
H18	0.7191	0.8823	0.6108	0.115*	
C19	0.6226 (14)	0.8790 (5)	0.4820 (4)	0.126 (2)	
H19	0.7670	0.9207	0.4848	0.152*	
C20	0.4389 (15)	0.8404 (5)	0.4029 (4)	0.1021 (19)	
H20	0.4556	0.8571	0.3524	0.122*	
C21	0.2354 (15)	0.7787 (6)	0.3985 (4)	0.129 (3)	
H21	0.1123	0.7510	0.3441	0.155*	
C22	0.2078 (11)	0.7562 (5)	0.4739 (4)	0.120 (2)	
H22	0.0653	0.7128	0.4695	0.144*	
F1	1.0623 (6)	0.9185 (2)	1.2596 (2)	0.1122 (11)	
F2	0.9669 (6)	1.0015 (2)	1.1873 (2)	0.1166 (11)	
F3	0.7503 (7)	0.9915 (2)	1.2909 (2)	0.1170 (11)	
N1	0.8220 (6)	0.7880 (2)	1.04998 (19)	0.0438 (8)	
O1	1.0137 (5)	0.7125 (2)	1.13247 (18)	0.0606 (8)	
O2	0.5759 (5)	0.77960 (19)	1.17672 (18)	0.0603 (7)	
O3	0.4658 (5)	0.87827 (19)	1.09346 (18)	0.0568 (7)	
O4	1.4169 (6)	0.4573 (2)	0.8761 (2)	0.0683 (8)	
O5	1.2311 (6)	0.4429 (2)	0.7298 (2)	0.0800 (10)	
O6	0.5276 (5)	0.8156 (2)	0.71026 (18)	0.0612 (8)	
O7	0.145 (5)	0.763 (2)	0.6537 (16)	0.108 (3)	0.244 (15)
O7'	0.1783 (13)	0.7010 (8)	0.6301 (5)	0.108 (3)	0.756 (15)
S1	0.65022 (19)	0.83949 (7)	1.13126 (7)	0.0482 (3)	

### Atomic displacement parameters (Å^2^)

**Table:**

	*U*^11^	*U*^22^	*U*^33^	*U*^12^	*U*^13^	*U*^23^
C1	0.039 (2)	0.048 (2)	0.050 (2)	−0.0061 (18)	0.0014 (18)	0.025 (2)
C2	0.038 (2)	0.039 (2)	0.056 (2)	−0.0019 (18)	0.0043 (18)	0.030 (2)
C3	0.048 (2)	0.052 (2)	0.053 (2)	0.000 (2)	0.0002 (19)	0.032 (2)
C4	0.045 (2)	0.044 (2)	0.067 (3)	0.002 (2)	0.005 (2)	0.034 (2)
C5	0.066 (3)	0.035 (2)	0.061 (3)	0.007 (2)	0.014 (2)	0.020 (2)
C6	0.066 (3)	0.051 (3)	0.056 (3)	0.007 (2)	0.001 (2)	0.029 (2)
C7	0.042 (2)	0.038 (2)	0.050 (2)	−0.0035 (17)	0.0004 (18)	0.0239 (19)
C8	0.042 (2)	0.045 (2)	0.051 (2)	−0.0021 (18)	0.0014 (17)	0.0287 (19)
C9	0.042 (2)	0.045 (2)	0.052 (2)	−0.0022 (18)	0.0057 (17)	0.028 (2)
C10	0.073 (3)	0.041 (2)	0.058 (2)	0.010 (2)	0.015 (2)	0.028 (2)
C11	0.078 (3)	0.057 (3)	0.075 (3)	0.020 (2)	0.020 (2)	0.045 (3)
C12	0.049 (2)	0.066 (3)	0.066 (3)	0.006 (2)	0.007 (2)	0.047 (2)
C13	0.054 (2)	0.056 (3)	0.051 (2)	0.005 (2)	0.0006 (18)	0.032 (2)
C14	0.077 (3)	0.056 (3)	0.051 (3)	−0.004 (3)	−0.001 (2)	0.022 (2)
C15	0.085 (3)	0.053 (3)	0.087 (3)	0.019 (3)	0.018 (3)	0.030 (3)
C16	0.059 (3)	0.090 (4)	0.064 (3)	−0.003 (3)	0.001 (2)	0.043 (3)
C17	0.074 (3)	0.064 (3)	0.047 (2)	0.008 (3)	0.008 (2)	0.025 (2)
C18	0.111 (4)	0.105 (4)	0.069 (3)	−0.026 (4)	−0.008 (3)	0.051 (3)
C19	0.154 (6)	0.143 (6)	0.100 (4)	−0.025 (5)	0.007 (4)	0.087 (5)
C20	0.149 (6)	0.111 (5)	0.069 (4)	0.035 (5)	0.033 (4)	0.054 (4)
C21	0.135 (6)	0.183 (7)	0.068 (4)	−0.015 (6)	−0.020 (4)	0.071 (5)
C22	0.110 (5)	0.165 (6)	0.082 (4)	−0.038 (4)	−0.019 (3)	0.075 (4)
F1	0.107 (2)	0.084 (2)	0.106 (2)	−0.0041 (18)	−0.0486 (18)	0.0218 (18)
F2	0.139 (3)	0.087 (2)	0.103 (2)	−0.0575 (19)	−0.0091 (19)	0.0465 (19)
F3	0.143 (3)	0.082 (2)	0.088 (2)	0.006 (2)	0.039 (2)	−0.0010 (18)
N1	0.0518 (19)	0.0442 (18)	0.0435 (17)	0.0074 (16)	0.0085 (14)	0.0269 (15)
O1	0.0642 (18)	0.0707 (19)	0.0566 (17)	0.0096 (15)	0.0040 (14)	0.0384 (16)
O2	0.0625 (18)	0.0626 (18)	0.0740 (18)	0.0030 (14)	0.0240 (14)	0.0465 (16)
O3	0.0490 (16)	0.0614 (18)	0.0709 (17)	0.0112 (14)	0.0095 (13)	0.0386 (15)
O4	0.076 (2)	0.0545 (18)	0.078 (2)	0.0220 (17)	0.0068 (16)	0.0306 (17)
O5	0.114 (3)	0.063 (2)	0.0711 (19)	0.037 (2)	0.0161 (19)	0.0302 (18)
O6	0.0621 (18)	0.0702 (19)	0.0643 (17)	−0.0017 (15)	−0.0047 (14)	0.0483 (16)
O7	0.111 (3)	0.111 (7)	0.080 (4)	−0.066 (5)	−0.018 (3)	0.049 (5)
O7'	0.111 (3)	0.111 (7)	0.080 (4)	−0.066 (5)	−0.018 (3)	0.049 (5)
S1	0.0451 (6)	0.0471 (6)	0.0553 (6)	−0.0005 (5)	0.0072 (5)	0.0271 (5)

### Geometric parameters (Å, °)

**Table:**

C1—O1	1.211 (4)	C13—H13B	0.9700
C1—N1	1.456 (4)	C14—F3	1.294 (5)
C1—C2	1.472 (5)	C14—F1	1.313 (5)
C2—C7	1.403 (5)	C14—F2	1.315 (5)
C2—C3	1.414 (5)	C14—S1	1.829 (4)
C3—C4	1.361 (5)	C15—O4	1.425 (5)
C3—H3	0.9300	C15—O5	1.435 (5)
C4—C5	1.375 (5)	C15—H15A	0.9700
C4—O4	1.379 (4)	C15—H15B	0.9700
C5—C6	1.373 (5)	C16—O7	1.14 (3)
C5—O5	1.377 (4)	C16—O7'	1.287 (9)
C6—C7	1.402 (5)	C16—O6	1.328 (5)
C6—H6	0.9300	C16—C17	1.464 (6)
C7—C8	1.521 (5)	C17—C22	1.362 (6)
C8—C9	1.526 (5)	C17—C18	1.367 (6)
C8—C13	1.527 (4)	C18—C19	1.375 (6)
C8—H8	0.9800	C18—H18	0.9300
C9—C10	1.501 (5)	C19—C20	1.367 (8)
C9—N1	1.518 (4)	C19—H19	0.9300
C9—H9	0.9800	C20—C21	1.335 (8)
C10—C11	1.318 (5)	C20—H20	0.9300
C10—H10	0.9300	C21—C22	1.370 (7)
C11—C12	1.479 (6)	C21—H21	0.9300
C11—H11	0.9300	C22—H22	0.9300
C12—O6	1.453 (4)	N1—S1	1.625 (3)
C12—C13	1.517 (5)	O2—S1	1.422 (2)
C12—H12	0.9800	O3—S1	1.420 (2)
C13—H13A	0.9700		
			
O1—C1—N1	120.4 (3)	H13A—C13—H13B	108.2
O1—C1—C2	124.1 (3)	F3—C14—F1	108.3 (4)
N1—C1—C2	115.5 (3)	F3—C14—F2	108.1 (4)
C7—C2—C3	121.1 (3)	F1—C14—F2	106.6 (4)
C7—C2—C1	122.3 (3)	F3—C14—S1	110.0 (3)
C3—C2—C1	116.5 (3)	F1—C14—S1	112.2 (3)
C4—C3—C2	117.7 (3)	F2—C14—S1	111.4 (3)
C4—C3—H3	121.2	O4—C15—O5	107.9 (3)
C2—C3—H3	121.2	O4—C15—H15A	110.1
C3—C4—C5	121.2 (4)	O5—C15—H15A	110.1
C3—C4—O4	128.8 (3)	O4—C15—H15B	110.1
C5—C4—O4	110.0 (4)	O5—C15—H15B	110.1
C6—C5—C4	122.9 (4)	H15A—C15—H15B	108.4
C6—C5—O5	127.3 (4)	O7—C16—O7'	44.0 (14)
C4—C5—O5	109.8 (3)	O7—C16—O6	114.1 (11)
C5—C6—C7	117.6 (3)	O7'—C16—O6	120.4 (4)
C5—C6—H6	121.2	O7—C16—C17	118.6 (12)
C7—C6—H6	121.2	O7'—C16—C17	124.5 (5)
C6—C7—C2	119.5 (3)	O6—C16—C17	114.2 (4)
C6—C7—C8	122.4 (3)	C22—C17—C18	117.0 (4)
C2—C7—C8	118.0 (3)	C22—C17—C16	120.8 (5)
C7—C8—C9	107.3 (3)	C18—C17—C16	122.2 (4)
C7—C8—C13	115.1 (3)	C17—C18—C19	121.3 (5)
C9—C8—C13	111.2 (3)	C17—C18—H18	119.4
C7—C8—H8	107.7	C19—C18—H18	119.4
C9—C8—H8	107.7	C20—C19—C18	119.8 (6)
C13—C8—H8	107.7	C20—C19—H19	120.1
C10—C9—N1	116.7 (3)	C18—C19—H19	120.1
C10—C9—C8	113.9 (3)	C21—C20—C19	119.6 (5)
N1—C9—C8	106.5 (3)	C21—C20—H20	120.2
C10—C9—H9	106.3	C19—C20—H20	120.2
N1—C9—H9	106.3	C20—C21—C22	120.1 (6)
C8—C9—H9	106.3	C20—C21—H21	119.9
C11—C10—C9	122.0 (4)	C22—C21—H21	119.9
C11—C10—H10	119.0	C17—C22—C21	122.1 (6)
C9—C10—H10	119.0	C17—C22—H22	118.9
C10—C11—C12	124.4 (4)	C21—C22—H22	118.9
C10—C11—H11	117.8	C1—N1—C9	114.8 (3)
C12—C11—H11	117.8	C1—N1—S1	119.7 (2)
O6—C12—C11	108.2 (3)	C9—N1—S1	125.4 (2)
O6—C12—C13	108.5 (3)	C4—O4—C15	105.8 (3)
C11—C12—C13	111.4 (3)	C5—O5—C15	105.8 (3)
O6—C12—H12	109.6	C16—O6—C12	118.9 (3)
C11—C12—H12	109.6	O3—S1—O2	120.49 (17)
C13—C12—H12	109.6	O3—S1—N1	109.04 (14)
C12—C13—C8	110.1 (3)	O2—S1—N1	110.45 (15)
C12—C13—H13A	109.6	O3—S1—C14	105.4 (2)
C8—C13—H13A	109.6	O2—S1—C14	105.50 (19)
C12—C13—H13B	109.6	N1—S1—C14	104.7 (2)
C8—C13—H13B	109.6		
			
O1—C1—C2—C7	166.9 (4)	C22—C17—C18—C19	2.2 (9)
N1—C1—C2—C7	−13.0 (5)	C16—C17—C18—C19	−179.4 (5)
O1—C1—C2—C3	−11.4 (5)	C17—C18—C19—C20	−0.3 (10)
N1—C1—C2—C3	168.7 (3)	C18—C19—C20—C21	−1.7 (10)
C7—C2—C3—C4	−0.7 (5)	C19—C20—C21—C22	1.7 (11)
C1—C2—C3—C4	177.6 (3)	C18—C17—C22—C21	−2.3 (9)
C2—C3—C4—C5	−0.5 (6)	C16—C17—C22—C21	179.3 (6)
C2—C3—C4—O4	177.5 (3)	C20—C21—C22—C17	0.3 (11)
C3—C4—C5—C6	1.1 (6)	O1—C1—N1—C9	160.2 (3)
O4—C4—C5—C6	−177.2 (3)	C2—C1—N1—C9	−19.9 (4)
C3—C4—C5—O5	178.6 (3)	O1—C1—N1—S1	−23.1 (5)
O4—C4—C5—O5	0.3 (4)	C2—C1—N1—S1	156.9 (3)
C4—C5—C6—C7	−0.5 (6)	C10—C9—N1—C1	−172.6 (3)
O5—C5—C6—C7	−177.5 (4)	C8—C9—N1—C1	59.0 (4)
C5—C6—C7—C2	−0.7 (5)	C10—C9—N1—S1	10.9 (5)
C5—C6—C7—C8	178.2 (3)	C8—C9—N1—S1	−117.6 (3)
C3—C2—C7—C6	1.3 (5)	C3—C4—O4—C15	176.5 (4)
C1—C2—C7—C6	−176.9 (3)	C5—C4—O4—C15	−5.4 (4)
C3—C2—C7—C8	−177.7 (3)	O5—C15—O4—C4	8.3 (4)
C1—C2—C7—C8	4.1 (5)	C6—C5—O5—C15	−177.7 (4)
C6—C7—C8—C9	−143.5 (3)	C4—C5—O5—C15	4.9 (4)
C2—C7—C8—C9	35.4 (4)	O4—C15—O5—C5	−8.2 (4)
C6—C7—C8—C13	−19.3 (5)	O7—C16—O6—C12	43.6 (19)
C2—C7—C8—C13	159.7 (3)	O7'—C16—O6—C12	−5.8 (8)
C7—C8—C9—C10	165.4 (3)	C17—C16—O6—C12	−175.5 (3)
C13—C8—C9—C10	38.8 (4)	C11—C12—O6—C16	−140.0 (4)
C7—C8—C9—N1	−64.5 (3)	C13—C12—O6—C16	99.0 (4)
C13—C8—C9—N1	168.9 (3)	C1—N1—S1—O3	−158.2 (3)
N1—C9—C10—C11	−136.4 (4)	C9—N1—S1—O3	18.2 (3)
C8—C9—C10—C11	−11.5 (5)	C1—N1—S1—O2	−23.6 (3)
C9—C10—C11—C12	3.9 (7)	C9—N1—S1—O2	152.7 (3)
C10—C11—C12—O6	−143.0 (4)	C1—N1—S1—C14	89.5 (3)
C10—C11—C12—C13	−23.8 (6)	C9—N1—S1—C14	−94.2 (3)
O6—C12—C13—C8	169.2 (3)	F3—C14—S1—O3	63.8 (4)
C11—C12—C13—C8	50.1 (4)	F1—C14—S1—O3	−175.5 (3)
C7—C8—C13—C12	179.0 (3)	F2—C14—S1—O3	−56.1 (4)
C9—C8—C13—C12	−58.8 (4)	F3—C14—S1—O2	−64.7 (4)
O7—C16—C17—C22	−39.5 (19)	F1—C14—S1—O2	56.0 (4)
O7'—C16—C17—C22	12.2 (10)	F2—C14—S1—O2	175.4 (3)
O6—C16—C17—C22	−178.6 (5)	F3—C14—S1—N1	178.7 (3)
O7—C16—C17—C18	142.1 (18)	F1—C14—S1—N1	−60.6 (4)
O7'—C16—C17—C18	−166.1 (8)	F2—C14—S1—N1	58.8 (4)
O6—C16—C17—C18	3.0 (7)		

### Hydrogen-bond geometry (Å, °)

**Table:**

*D*—H···*A*	*D*—H	H···*A*	*D*···*A*	*D*—H···*A*
C3—H3···O4^i^	0.93	2.51	3.341 (5)	149
C15—H15A···O2^ii^	0.97	2.48	3.202 (5)	131

Symmetry codes: (i) −*x*+3, −*y*+1, −*z*+2; (ii) −*x*+2, −*y*+1, −*z*+2.
